# The effect of musicality on language recovery after awake glioma surgery

**DOI:** 10.3389/fnhum.2022.1028897

**Published:** 2023-01-10

**Authors:** Pablo R. Kappen, Jan van den Brink, Johannes Jeekel, Clemens M. F. Dirven, Markus Klimek, Marike Donders-Kamphuis, Christa S. Docter-Kerkhof, Saskia A. Mooijman, Ellen Collee, Rishi D. S. Nandoe Tewarie, Marike L. D. Broekman, Marion Smits, Arnaud J. P. E. Vincent, Djaina Satoer

**Affiliations:** ^1^Department of Neurosurgery, Erasmus University Medical Center, Rotterdam, Netherlands; ^2^Department of Neuroscience, Erasmus University Medical Center, Rotterdam, Netherlands; ^3^Department of Anesthesiology, Erasmus University Medical Center, Rotterdam, Netherlands; ^4^Department of Speech and Language Pathology, Haaglanden Medisch Centrum, The Hague, Netherlands; ^5^Department of Neurosurgery, Haaglanden Medisch Centrum, The Hague, Netherlands; ^6^Department of Neurosurgery, Leiden University Medical Center, Leiden, Netherlands; ^7^Department of Radiology & Nuclear Medicine, Erasmus University Medical Center, Rotterdam, Netherlands; ^8^Medical Delta, Delft, Netherlands; ^9^Brain Tumor Center, Erasmus MC Cancer Institute, Rotterdam, Netherlands

**Keywords:** music, neuro-oncology, neuroplasticity, corpus callosum, aphasia, brain tumors

## Abstract

**Introduction:**

Awake craniotomy is increasingly used to resect intrinsic brain tumors while preserving language. The level of musical training might affect the speed and extend of postoperative language recovery, as increased white matter connectivity in the corpus callosum is described in musicians compared to non-musicians.

**Methods:**

In this cohort study, we included adult patients undergoing treatment for glioma with an awake resection procedure at two neurosurgical centers and assessed language preoperatively (T1) and postoperatively at three months (T2) and one year (T3) with the Diagnostic Instrument for Mild Aphasia (DIMA), transferred to *z*-scores. Moreover, patients’ musicality was divided into three groups based on the Musical Expertise Criterion (MEC) and automated volumetric measures of the corpus callosum were conducted.

**Results:**

We enrolled forty-six patients, between June 2015 and September 2021, and divided in: group A (non-musicians, *n* = 19, 41.3%), group B (amateur musicians, *n* = 17, 36.9%) and group C (trained musicians, *n* = 10, 21.7%). No significant differences on postoperative language course between the three musicality groups were observed in the main analyses. However, a trend towards less deterioration of language (mean/SD *z*-scores) was observed within the first three months on the phonological domain (A: −0.425/0.951 vs. B: −0.00100/1.14 vs. C: 0.0289/0.566, *p*-value = 0.19) with a significant effect between non-musicians vs. instrumentalists (A: −0.425/0.951 vs. B + C: 0.201/0.699, *p* = 0.04). Moreover, a non-significant trend towards a larger volume (mean/SD cm^3^) of the corpus callosum was observed between the three musicality groups (A: 6.67/1.35 vs. B: 7.09/1.07 vs. C: 8.30/2.30, *p* = 0.13), with the largest difference of size in the anterior corpus callosum in non-musicians compared to trained musicians (A: 3.28/0.621 vs. C: 4.90/1.41, *p* = 0.02).

**Conclusion:**

With first study on this topic, we support that musicality contributes to language recovery after awake glioma surgery, possibly attributed to a higher white matter connectivity at the anterior part of the corpus callosum. Our conclusion should be handled with caution and interpreted as hypothesis generating only, as most of our results were not significant. Future studies with larger sample sizes are needed to confirm our hypothesis.

## Introduction

Awake craniotomy is increasingly used to resect intrinsic brain tumors (specifically for diffuse low-grade gliomas) while preserving language. This technique has improved over time, with the development of intraoperative protocols for awake tumor resection (De Witte et al., 2015). Despite these improvements, intraoperative mapping and language testing do not always ensure complete maintenance of the patient’s linguistic abilities. Due to slow tumor growth, diffuse low grade glioma patients typically suffer from mild aphasia preoperatively which often temporarily deteriorates after tumor resection (Duffau et al., 2003; Satoer et al., 2014). In the year after surgery, most patients recover to their baseline level whereas others remain to suffer from this further language decline in the long-term (Satoer et al., 2014). This can be attributed to differences in neuroplasticity in language networks, but it is unclear which factors and to what degree these affect postoperative language recovery (Kiran and Thompson, 2019).

The literature suggests that musical training might affect the course of postoperative language recovery (Merrett et al., 2013). Both language and music require complex hierarchical processing systems that share features, such as pitch, rhythm, timbre, and syntactic structure (Besson and Schön, 2001). Recent fMRI data suggested that some brain regions, associated with language functioning (e.g., Broca and Wernicke’s areas), are also activated during music processing (Maess et al., 2001; Levitin and Menon, 2003; Abrams et al., 2011).

Higher degree of organization of language structures between lobes (i.e., frontal and temporal) or hemispheres through the corpus callosum have been described in musicians (Schlaug et al., 1995a,^2009^). This has provided ground for music-induced language therapy, such as Melodic Intonation Therapy (MIT), in patients with severe aphasia (Schlaug et al., 1995a,^2009^; Omigie and Samson, 2014).

Some experimental studies show that musical training can improve language function (in a so-called transfer of learning) in healthy participants (Besson and Schön, 2001). However, there is currently no evidence in the literature to support the hypothesis that musical training-related brain changes might also have a beneficial effect on language following brain surgery (Omigie and Samson, 2014).

Hence, we conducted a study in which we hypothesize a better recovery of language in musical patients after awake glioma surgery as compared to non-musical patients. Moreover, we hypothesize that this possible beneficial effect may be explained by contralateral compensation through the corpus callosum.

## Materials and methods

### Study population

The consecutively included cohort consisted of adult patients, who underwent an awake resection between June 2015 and September 2021 at the Erasmus MC, University Medical Center Rotterdam (EMC) or at the Haaglanden Medisch Centrum the Hague (HMC), and received an extensive language assessment before (baseline; T1) and at least one time point after surgery (3 months; T2 and/or 1 year; T3). These centers consider awake surgery in case of left-sided tumors, right-sided tumors with left handedness or involvement of the sensory-motor regions or in case of prior speech deficits with or without language location confirmed by functional fMRI. Moreover, an awake craniotomy procedure is only considered if we deem this feasible for the particular patient. Patients that were operated for a recurrent glioma, non-native Dutch speakers (defined as unfamiliar with the Dutch language before the age of 8 years), patients known with neurodegenerative diseases affecting language (e.g., dementia) or with a WHO grade 4 astrocytoma or glioblastoma, were excluded. Patients were additionally excluded for the volumetric analysis in case of tumor involvement in the corpus callosum.

### Study design and data extraction

Data on musicality were prospectively collected through a questionnaire and retrospectively complemented with available language and clinical data.

#### Musicality

The Musical Expertise Criteria (MEC) are based on years of musical training and intensity and define a musician based on the “six-year rule” of training (Lamont, 2002; Lotze et al., 2003; Weijkamp and Sadataka, 2017; Zhang et al., 2018). A questionnaire was developed, based on the MEC, in which points were allocated to the patient, leading to final group formation; non-musicians (group A), amateur musicians (group B), and trained musicians (group C, Supplementary Appendix A). Additional information on musicality was assessed such as the onset age of playing the instrument/vocals, type of instrument and whether music was played after the operation.

#### Linguistic data

Language data were retrospectively extracted as language was already monitored with the Diagnostic Instrument for Mild Aphasia (DIMA) as part of standard of clinical care at baseline (T1) and at least one time point after surgery (3 months; T2 and/or 1 year; T3) (Satoer et al., 2022). The DIMA is a tool, developed and validated in Dutch to evaluate suspected mild aphasia in patients with glioma (Satoer et al., 2022). It consists of six subtests and assesses language production and comprehension in the following linguistic domains: phonology, semantics and (morpho-) syntax. Moreover, data from a non-linguistic cognitive test for visual attention and mental flexibility (Trail Making Test/TMT A, B, and BA) were extracted (Llinas-Regla et al., 2017).

#### Clinical data

Clinical data were extracted consisting of demographic data (age, sex, education years and level based on the Verhage scale, handedness), disease specifications (histopathology, localization), and treatment specifications (completeness resection, complications, adjuvant treatment) (Verhage, 1964a,b; Reitan, 1989).

#### Volumetry

To measure the size of the corpus callosum we analyzed the most recent structural brain magnetic resonance imaging (MRI: 1.5 or 3.0 Tesla GE Healthcare) before the awake craniotomy, using <1.0 mm slide with T1 weighted imaging parameters. Two researchers (P.K./J.B.), blinded for the outcome on musicality at the time of measurement, first divided the corpus callosum in seven subregions according to the Witelson classification (Witelson and McCulloch, 1991). Afterward, volumes (in cubic centimeters/cm^3^) for each subregion were measured with Brainlab’s Synthetic Tissue Model (Brainlab Digital OR, München, Germany). In this model each anatomical structure is first detected and then adapted to a gray-scale image model. Tissue-class specific gray value simulation is compared with meta information from datasets and afterward quantitatively and qualitatively validated. This software is CE marked and already widely applied for guidance during neurosurgical procedures. Sub-group analyses were conducted for sex and onset/duration of musical training, as differences in corpus callosum volumes have been described in these factors (Schlaug et al., 1995a; Lee et al., 2003). For the volume lesion analysis we used the pre-operative coronal, sagittal and transversal T2 weighted FLAIR MRI images and conducted volumetric analysis with Brainlabs’ smart brush (see Supplementary Appendix B for further Technical Background).

### Statistical analysis

The raw DIMA and TMT scores (A, B, and BA) were transferred into *z*-scores corrected for age and years of education, in order to facilitate comparisons. For each of the corpus callosum subregions, an inter-rater agreement was calculated with the interclass correlation coefficient (ICC). Corpus callosum region volumes were compared between groups based on the raw (cm^3^) and corrected measurements (corpus callosum volume divided by total brain volume).

The three musicality groups and language or corpus callosum volumes were visually evaluated and statistically compared with an ANOVA in case of parametric data and a Kruskal–Wallis test in case of non-parametric data. Normality was tested with the Shapiro–Wilk test. Correlations between musical training, size of corpus callosum, and course of postoperative language were conducted with the Pearson’s product-moment correlation. For all analyses significance (*p*-value, significant in case of 0.05 or less), and for correlation coefficient (*r*), degrees of freedom (df) were illustrated.

We were unable to conduct *a priori* sample size calculation, as we were unsure which effect size was expected as this is the first study evaluating the effects of musicality on language recovery after awake glioma surgery. Hence, achieved power was computed (1-β) on *post-hoc* analyses in case of visually observed non-significant outcomes using G*Power version 3.1 (Faul et al., 2009). All other statistical analyses were conducted using R (version 4.1.1).

## Results

### Musicality and demographic data

We consecutively included 46 patients, in the period between June 2015 and September 2021, at the EMC (*n* = 39) and HMC (*n* = 7). Patients were divided into three groups based on musicality: non-musician (A: *n* = 19, 41.3%), amateur musician (B: *n* = 17, 36.9%), and trained musicians (C: *n* = 10, 21.7%).

The mean (SD) age at the time of craniotomy was 39.6 (12.0) years; 18 women (39.1%) and 40 (87.0%) right-handed patients (Table 1). Higher education level was observed in 24 (52.2%) patients, with mean (SD) number of years of education of 14.8 (2.47). Gross total resection of the tumor was achieved in 20 (56.5%) patients. Intra-operative complications were reported in 4 (8.7%) patients; one patient had an arterial bleeding which was coagulated and three other patients had intra-operative seizures during mapping.

**TABLE 1:** Baseline characteristics.

	A. Non-musician *N* = 19	B. Amateur musician *N* = 17	C. Trained musician *N* = 10	*P*-Value
**Demographic data**				
Age (mean/SD)^1^	38.8 (11.7)	40.3 (14.3)	39.6 (9.31)	0.92
Female sex (*n*/%)	9 (47.4%)	6 (37.5%)	3 (27.3%)	0.61
Higher education (*n*/%)^2^	6 (31.6%)	12 (70.6%)	6 (60.0%)	0.06
Education years (mean/SD)	14.1 (2.27)	15.1 (2.42)	15.5 (2.83)	0.17
Right handedness	16 (84.2%)	14 (82.4%)	10 (100%)	0.38
**Disease and surgical specifics**				
High grade tumor (*n*/%)	1 (5.3%)	5 (29.4%)	1 (10.0%)	0.12
Right sided localization (*n*/%)	5 (26.3%)	7 (41.2%)	8 (80.0%)	0.02
Lesion volume (mean/SD cm^3^)	31.4 (19.2)	49.2 (31.7)	36.5 (28.0)	0.20
Gross total resection (*n*/%)	7 (36.8%)	8 (47.1%)	5 (50.0%)	0.74
Intra-operative complications (*n*/%)	1 (5.3%)	1 (5.9%)	2 (20.0%)	0.36
Adjuvant treatment (*n*/%)^3^	5 (26.3%)	9 (52.9%)	2 (20.0%)	0.13
**Cognitive function^4^**				
TMT A (mean/SD)	0.889 (1.63)	1.24 (1.64)	0.810 (1.68)	0.63
TMT B (mean/SD)	0.611 (0.918)	0.318 (1.73)	0.660 (0.862)	0.97
TMT BA (mean/SD)	0.358 (1.21)	0.288 (1.19)	0.160 (0.937)	0.97
**Musical specifications^5^**				
Main instrument	–			
Singing		4 (23.5%)	2 (20%)	
Instrument	–	15 (88.2%)	10 (100%)	0.46
Start age main instrument (mean/SD)	–	13.1 (8.44)	12.0 (4.59)	0.78
Start instrument under 10 years (n/%)		10 (58.8%)	3 (30.0%)	0.15
Total hours of playing (mean/SD)^6^	–	535 (743)	5020 (3890)	< 0.001

^1^Age at awake craniotomy.

^2^Finished high level secondary education or university degree.

^3^Received adjuvant therapy, including chemotherapy (i.e., temozolomide) or radiotherapy, until 1 year after surgery.

^4^Trail making test; z values.

^5^*P*-values were calculated between the amateur and trained musicians. +Mean hours per day × years (×365) playing.

Adjuvant therapy within one year was administered in 16 (65.2%) patients. Histopathology revealed WHO grade 2 glioma in 39 (84.8%) patients and tumor localization was right-sided in 20 (43.5%) patients. None of the baseline characteristics differed significantly among groups, except for right sided tumor localization, which was more common in group C (*p* = 0.02).

Trail Making Test (mean/SD *z*-scores) were 0.289/1.13–1.00/1.62 (average to high average) and were similar between the three groups. In musical patients, the mean/SD age of starting to play an instrument was 13.1/8.44 years (group B) and 12.0/4.59 years (group C), with a mean/SD total of hours of playing music of 535/743 (group B) and 5020/3890 (group C).

### Primary outcome: Musicality vs. language

**FIGURE 1:**
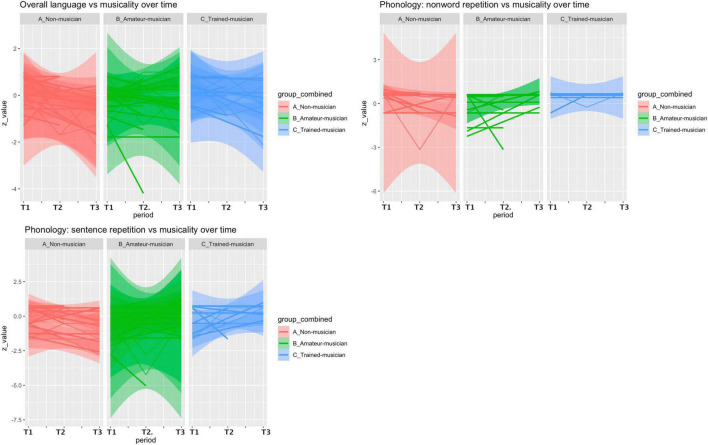
Postoperative language course vs. musicality. An overall decrease of language performance (mean/SD *z*-value) was observed within the first three months (T1 vs. T2) in our included cohort (*n* = 44, –0.255/0.966) but patients with more musical experience tended to recover better on the non-word repetition subtest (phonologic domain) (A: –0.425/0.951 vs. B: –0.001/1.14 vs. C: 0.028/0.566, *p* = 0.19) and the sentence repetition subtest (phonologic domain) (A: –0.202/0.683 vs. B: 0.036/1.92 vs. C: 0.125/1.32, *p* = 0.446).

Our main analyses comparing musicality and postoperative course of language were not statistically significant (Figure 1, Table 2, and Supplementary Appendix C). An overall decrease of language performance (mean/SD *z*-value) was observed within the first three months (T1 vs. T2) in our included cohort (*n* = 44, −0.255/0.966, Table 2), which was not different between the three groups (A: −0.411/0.865 vs. B: −0.0947/1.18 vs. C: −0.227/0.779, *p* = 0.45).

**TABLE 2:** DIMA scores vs. musicality.

	Baseline (T1) vs. after 3 months (T2)			After 3 months (T2) vs. after 1 year (T3)		
	**A. Non-musician**	**B. Amateur musician**	**C. Trained musician**	**A. Non-musician**	**B. Amateur musician**	**C. Trained musician**
**Overall**						
DIMA	−0.411 (0.865)	−0.0947 (1.18)	−0.227 (0.779)	−0.260 (1.07)	−0.265 (0.818)	−0.178 (1.19)
Days*	68.3 (18.8)	73.6 (33.4)	67.2 (39.1)	347 (77.3)	297 (110)	353 (70.5)
**Phonology**						
Word repetition	−0.293 (1.20)	−0.510 (1.86)	0 (0)	0.568 (1.71)^□^	0.393 (2.30)^□^	0 (0)^□^
Compound repetition	−0.308 (1.83)	−0.805 (1.66)	−0.214 (0.642)	0 (1.45)	−0.224 (0.806)	0.385 (0.861)
Non-word repetition	−0.425 (0.951)**^Δ^**	−0.00100 (1.14)**^Δ^**	0.0289 (0.566)**^Δ^**	0.279 (1.63)	0.0768 (0.582)	0.199 (0.445)
Sentence repetition	−0.202 (0.683)**^Δ^**	0.0359 (1.92)**^Δ^**	0.125 (1.32)**^Δ^**	−0.318 (0.631)	0.426 (1.59)	0.226 (0.505)
**Semantic**						
Semantic tests	−0.470 (1.32)	−0.225 (1.20)	−0.234 (0.703)	−0.234 (1.27)	0.162 (1.04)	0 (0)
**Syntaxis**						
Sentence completion	0.0316 (2.09)^Δ^	−0.0484 (1.46)^Δ^	−0.531 (1.45)^Δ^	−1.09 (2.46)	−1.37 (1.96)	−1.01 (4.43)

All values are mean (SD) Z scores, calculated from a healthy population (*n* = 211) based on age (cutoff 55 years) and Education years (cutoff 12 years). T1; baseline/before surgery, T2; 3 months after surgery, T3; 1 year after surgery. *Mean (SD) days from craniotomy to T2/T3. ^Δ^Beneficial trend observed between musicality and language. ^□^Detrimental trend observed between musicality and language.

Within the first 3 months (T1 vs. T2), patients with more musical experience tended to recover better in the phonologic domain on the non-word repetition subtest (A: −0.425/0.951 vs. B: −0.001/1.14 vs. C: 0.028/0.566, *p* = 0.19, effect size: 0.233, 1-β = 0.26) and the sentence repetition subtest (A: −0.202/0.683 vs. B: 0.036/1.92 vs. C: 0.125/1.32, *p* = 0.44, effect size = 0.09, 1-β = 0.08), and recover less on the syntactic domain in the sentence completion subtest (A: 0.031/2.09 vs. B: −0.048/0.46 vs. C: −0.531/1.45, *p* = 0.86, effect size = 0.127, 1-β = 0.11). However, these differences were not significant. In the period of 3 months to 1 year (T2 vs. T3) a decrease of language performance (*z*-value mean/SD) was observed (*n* = 27, −0.246/0.947), which was not different between the groups (A: −0.178/1.19 vs. B: −0.265/0.818 vs. C: −0.260/1.07, *p* = 0.90), but a beneficial effect of non-musicality was found in the word repetition subtest (phonologic domain, A: 0/0 vs. B: 0.393/2.30 vs. C: 0.568/1.71, *p* = 0.86, effect size = 0.19, 1-β = 0.18). *Post-hoc* analyses revealed a maximum achieved power (1-β) of 26%.

Sub-analyses within the musicians (B and C), comparing instrument players (*n* = 21) with singers (*n* = 7) revealed worse language performance of singers within the first 3 months (0.0428/0.837 vs. −0.729/1.44, *p* = 0.21), in the compound word repetition subtest (phonologic domain, −0.248/0.776 vs. −1.77/2.33, *p* = 0.03) and the semantic subtest (0/0.968 vs. −0.990/0.949, *p* = 0.01). Excluding singers from the main analyses revealed a significant effect within the first three months (T1 vs. T2) on the non-word repetition subtest (phonologic domain) when comparing non-musicians vs. instrumentalist musicians (A: −0.425/0.951 vs. B and C: 0.201/0.699, *p* = 0.039).

### Secondary outcome: Musicality vs. corpus callosum

**FIGURE 2:**
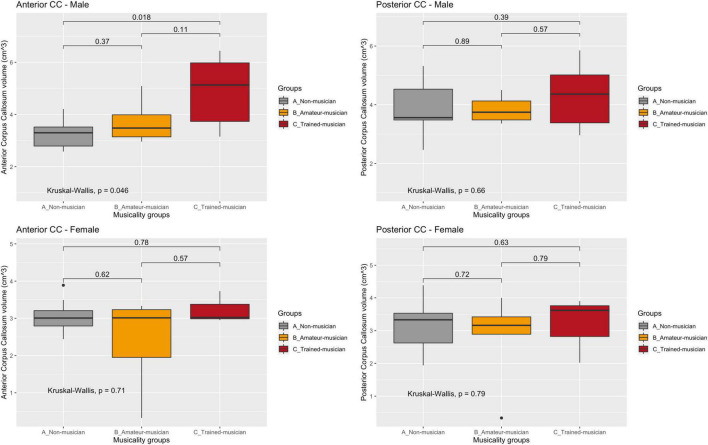
Corpus callosum regions vs. musicality per sex. CC, corpus callosum. Sub-analyses in sex and subregion revealed the largest trend in the anterior corpus callosum of male non- vs. trained musicians (A: 3.28/0.621 vs. C: 4.90/1.41, *p* = 0.05). No trend was observed in women nor in the posterior corpus callosum.

Volumetric corpus callosum measurements were obtained from 39 patients: inter-class correlation showed good to excellent inter-observer agreement (ICC = 0.77–0.99) for each corpus callosum region. No statistically significant difference was observed between the musicality groups and the corpus callosum volumes (Figure 2 and Table 3).

**TABLE 3:** Corpus callosum measurements vs. musicality.

	A. Non-musician *N* = 17	B. Amateur musician *N* = 13	C. Trained musician *N* = 9	*P*-Value
**Overall (*n* = 39)**				
Corpus callosum	6.67 (1.35)	7.09 (1.07)	8.30 (2.30)	0.13
Anterior corpus callosum	3.17 (0.551)	3.16 (1.12)	4.34 (1.41)	0.06
Posterior corpus callosum	3.49 (0.878)	3.42 (1.03)	3.93 (1.17)	0.52
**Male (*n* = 23)**				
Corpus callosum	7.08 (1.42)	7.54 (0.995)	9.23 (2.18)	0.12
Anterior corpus callosum	3.28 (0.621)	3.66 (0.723)	4.90 (1.41)	0.05
Posterior corpus callosum	3.79 (0.905)	3.83 (0.433)	4.30 (1.13)	0.66
**Female (*n* = 16)**				
Corpus callosum	6.21 (1.19)	6.36 (0.808)	6.44 (1.26)	0.13
Anterior corpus callosum	3.05 (0.468)	2.37 (1.27)	3.23 (0.432)	0.70
Posterior corpus callosum	3.16 (0.762)	2.76 (1.41)	3.18 (1.01)	0.78

All volume measures are in mean/SD cubic centimeter (cm^3^). Patients with tumor involvement in the corpus callosum were excluded. Anterior corpus callosum: rostrum, genu, rostral body and anterior body. Posterior corpus callosum: posterior body, isthmus and splenium.

A trend of effect of musicality on corpus callosum volume (mean/SD cm^3^) was observed (A: 6.67/1.35 vs. B: 7.09/1.07 vs. C: 8.30/2.30, *p* = 0.13) which diminished after correcting for total brain volume (A; 0.756/0.128 vs. B: 0.763/0.091 vs. C: 0.837/0.221, *p* = 0.63).

Sub-analyses in sex and subregion revealed the largest difference in the anterior corpus callosum of male non- vs. trained musicians (A: 3.28/0.621 vs. C: 4.90/1.41, *p* = 0.05). No trend was observed in women nor in the posterior corpus callosum. Size of corpus callosum (mean/SD cm^3^) was not significantly larger in patients that started playing their instrument before their tenth life year (7.33 vs. 7.84, *p* = 0.81).

**FIGURE 3:**
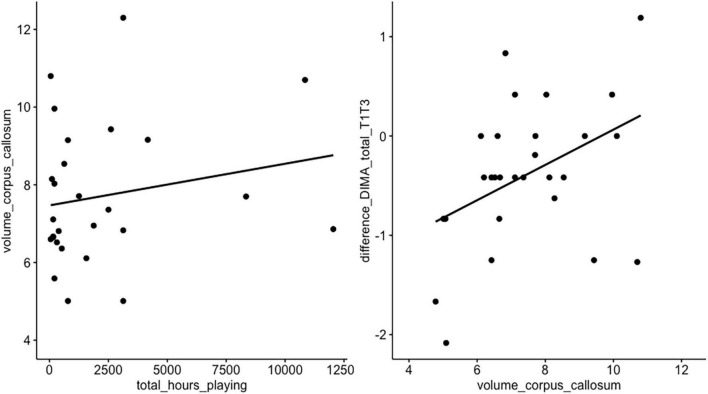
Correlation hours of playing vs. volume of corpus callosum vs. language. A linear correlation was visually observed, but not statistically confirmed, between volume of corpus callosum and postoperative language course (T1 vs. T3, *t* = 0.79, df = 22, *p*-value = 0.43) and between the total hours of playing and corpus callosum volume (*t* = 1.57, df = 18, *p*-value = 0.13).

A linear correlation was visually observed, but not statistically confirmed, between volume of corpus callosum and postoperative language course (T1 vs. T3, *t* = 0.79, df = 22, *p*-value = 0.43) and between the total hours of playing and corpus callosum volume (*t* = 1.57, df = 18, *p*-value = 0.13, Figure 3).

## Discussion

In this cohort study, we evaluated the effect of musicality on the course of post-operative language recovery following awake glioma surgery. We did not find a significant difference between musicality, corpus callosum size and postoperative course of language performance after awake glioma surgery in our main analysis. This could point into the direction that there is no correlation between musicality and language recovery. However, the lack of evidence could also be attributed to our limited sample size, as our power (1-β) concerning possible trends did not exceed 26%. Future studies with larger sample size could confirm our findings.

Although most findings did not reach significance, we did observe a significant beneficial effect, after excluding the vocal musicians, in two phonological subtests in patients with a musical background compared to non-musicians. The observed effect in our study related to musicality and phonology is not unexpected as the phonologic system and music share a common hierarchical structure (e.g., syllabic and grouping structure, prosody and melody). In the phonological subtests existing words and non-words had to be repeated, including a correct phonological form (including syllables and phonemes), stress patterns and pitch. Musical expertise increases sensitivity to pitch changes which allows musicians to detect subtle variations of pitch, rhythm, and harmony within musical phrases faster, and more accurately than non-musicians (Besson et al., 1994; Koelsch et al., 2002; Bidelman and Krishnan, 2010). This enhanced sensitivity to acoustic features might allow musicians to construct more elaborated perceptions of the speech signal, referred to as transfer effects, than non-musicians. This transfer effect was supported by a study showing that musicians were more sensitive than non-musicians to abstract phonological representations (consonant or vowel changes; e.g., baìn/zaìn) derived from the processing of acoustic parameters (Marie et al., 2011). This, in turn, can facilitate stages of speech processing, leading to higher scores on the phonologic language tests (Besson et al., 1994).

As our patients were asked to focus their attention during the test, one could argue that the beneficial results of musicians on language tests reflect a general effect of attention. However, data from a non-linguistic cognitive test for visual attention and mental flexibility (Trail Making Test) revealed average to high scores, which did not differ between groups. Moreover, electro-encephalogram studies tackled this issue by showing similar attention between both musicians and non-musicians while conducting several language tests (Courchesne et al., 1975; Squires et al., 1975; Escera et al., 2000). Our findings on phonology are clinically relevant as its prognostic relation to the quality of verbal communication at the long run were already demonstrated in aphasic patients after stroke (El Hachioui et al., 2013). The phonological subtests included, among other tests, non-word and sentence repetition; these two tests are important to address as they enable us to distinguish lexical from non-lexical processes. Additional to the classic theory, in which a lesion in the arcuate fasciculus leads to conduction aphasia (Wernicke, 1874), recent studies suggest that a word-repetition impairment may be explained by a “dual-route” model: a dorsal language stream which is dedicated to phonological processing (non-lexical: ability to link sound to articulation), and a ventral stream which is dedicated to semantic processing (lexical: linking sound to meaning) (Moritz-Gasser and Duffau, 2013). Therefore, it is important to monitor subtle changes in phonological production (e.g. word repetition) as an indicator for the overall quality of language processing (Sierpowska et al., 2017). Moreover, future language rehabilitation could be targeted at the phonological level in glioma patients with a musical background. The advantage of musicality on phonology between three months and one year was less prominent: restoration of language in the non-musical population may have reduced the beneficial effect of music induced alternative compensatory pathways for language recovery.

We did not expect the worse performance in the syntactic domain in the trained musicians compared to the non-musicians. In the literature a paradox is found on syntactic relations in music and language. Cases of dissociations have been described with impaired perception of harmonic relations in music (i.e., amusia) with no signs of aphasia or, inversely, language impairment with spared musical abilities (Griffiths et al., 1997; Peretz et al., 1997, 2003; Ayotte et al., 2000, 2002). On the other hand, associations have been described on neuroimaging studies showing early right anterior negativity (associated with harmonic processing) in Broca’s area (Maess et al., 2001). Patel (2003) tackled this paradox by proposing the ‘shared syntactic integration resource hypothesis’ in which linguistic and musical syntax share certain syntactic integration processes that apply over different domain-specific syntactic representations. The syntactic subtest involved completion of the sentence with words that would fit within the context, which also touches upon semantic performance. Therefore, the decrease of syntactic scores in the trained musician group may have been attributed to damage to domain-specific semantic representations rather than a problem with syntactic integration processes, which is expected to be enhanced in this sub-group.

A trend toward a larger corpus callosum, predominantly anteriorly, in trained musical patients compared to non-musical patients was observed. Anterior corpus callosum connects frontal structures; it has been suggested that the intense bimanual motor training of musicians, such as when playing a string instrument, could play an important role in the development of more and thicker myelinated transcallosal fibers (Schlaug et al., 1995a). This difference was mostly found in men, which confirms a prior study conducted by Lee et al. (2003). A pre-existing sex-based difference in brain symmetry was hypothesized by these researchers. Less brain symmetry, thus more functional lateralization, is observed in smaller corpus callosum volumes (Keenan et al., 2001). There are reports of women showing increased symmetry compared with men; the authors speculate that female musicians might not show a significant change in lateralization after repetitive bimanual motoric movement and therefore no effect on corpus callosum size (Dorion et al., 2001; Lee et al., 2003).

A paper on musicality and corpus callosum size reported an increased size for those musicians who commenced music training prior to seven years of age, which was confirmed by a number of papers since that time (Schlaug et al., 1995b; Ozturk et al., 2002; Lee et al., 2003). We were not able to assess this correlation as the trained musicians in our cohort started playing their instrument at an older age. There seemed to be a trend between the hours of musical training and the size of the corpus callosum, however this was not statistically confirmed. A longitudinal study investigating the influence of musical training on brain structure in children found a significant relationship between the amount of practice and the degree of structural change in the corpus callosum (Schlaug et al., 2005). Future studies should therefore not just consider when musicians start to train, but also how long and how much they train.

We observed a linear trend between the size of the corpus callosum, hours of musical training and postoperative language recovery. Musical patients may benefit from higher white matter connectivity in the corpus callosum, contributing to functional reorganization toward the contralateral side (Schlaug et al., 1995b; Amunts et al., 1997; Gaser and Schlaug, 2003; Lotze et al., 2003; Han et al., 2009; Hyde et al., 2009; Pantev and Herholz, 2011; Wu et al., 2013). Melodic Intonation Therapy (MIT), a rehabilitation technique using melodic intoning and rhythm to restore language, has been demonstrated to be beneficial in improved functional language in stroke patients with severe aphasia (Hurkmans et al., 2012). A current debate in the aphasia literature concerns whether this occurs due to contralateral hemisphere or ipsilateral perilesional compensation (Van Der Meulen et al., 2016). Presently, it is thought that contralateral activation occurs commonly in the post-acute phase, with a return to ipsilateral perilesional activation over the following months (Saur et al., 2006). Our results create some substantiation for contralateral compensation in the (sub-)acute phase through the corpus callosum. As our results were less clear after three months post-surgery, future studies could focus on the connectivity of the ipsilateral arcuate fasciculus and the role over time between musicians and non-musicians (Schlaug et al., 1995b; Amunts et al., 1997; Gaser and Schlaug, 2003; Lotze et al., 2003; Han et al., 2009; Hyde et al., 2009; Pantev and Herholz, 2011; Wu et al., 2013).

### Strengths and limitations

This is the first study supporting that musicality contributes to language recovery after awake glioma surgery possibly due to increased neuroplastic properties in language networks. This is relevant as increased knowledge on factors contributing to language recovery can be used in clinical practice to inform the patients on their prognosis and could even aid in the final decision-making when considering surgery. There are some limitations to discuss: the first and most important issue is that most of our findings were not statistically significant, which may be due to our limited sample size as our power did not exceed 26%. Our conclusions should therefore be interpreted to generate new hypotheses. Second, patients in the musical group had a higher level of education, which could have contributed to a better cognitive reserve, also described as ‘brain reserve capacity’. According to these models, the threshold of brain damage necessary to bring about a given deficit is more quickly reached in individuals with less cognitive training due to less brain reserve capacity (Gehring et al., 2011; Omigie and Samson, 2014; Stern and Barulli, 2019). However, we tend to tackle this by showing a similar cognitive level at baseline. Moreover, language scores were corrected for education level and age. Second, tumor in the right hemisphere was more often observed in the musical group which could be a confounding on language performance, considering that language is often lateralized in the left hemisphere. However, we argue that this does not influence our results as prior research found that hemispheric lateralization does not affect language performance on the DIMA scale in glioma patients (Mooijman et al., 2021; Satoer et al., 2022).

## Future studies

Future studies with a larger sample size should confirm our findings, and might be able to correct for the above-described confounding variables. Second, imaging techniques such as diffuse tensor imaging (DTI) and functional MRI (e.g., with language and musical (intonation) tests) before and after surgery could be linked to the course of postoperative language recovery to identify the role of contra- and ipsilateral compensation over time (Mendez Orellana et al., 2014). Last, quality of life questionnaires may be added to assess the true impact of subtle language differences between musical and non-musical patients after glioma surgery.

## Conclusion

This is the first study supporting that musicality contributes to language recovery after awake glioma surgery due to increased neuroplastic properties in language networks, especially in instrumentalists. This may be partly attributed to a higher white matter connectivity at the anterior part of the corpus callosum developed during repetitive bimanual musical training, which might have contributed to functional reorganization toward the contralateral side. Our conclusion should be handled with caution and interpreted as hypothesis generating only, as most of our results did not reach statistical significance. Future studies with larger sample sizes are needed to confirm our hypothesis.

## Data availability statement

The raw data supporting the conclusions of this article will be made available by the authors, without undue reservation.

## Ethics statement

Ethical approval for this study (MEC-2020-0351) was provided by the Ethical Committee of the Erasmus Medical Center, Rotterdam (Chairperson Prof. Dr. H. W. Tilanus) on the 23rd of March 2020 and of the Haaglanden Medical Center (MEC 2021-055, Chairperson Dr. D. Horbach) on the 6th of July 2021. The patients/participants provided their written informed consent to participate in this study.

## Authors contributions

PK and DS conceived the study idea, interpreted the data, and wrote the first draft of the manuscript. PK coordinated the research protocol. PK and JB extracted the data and analyzed the radiologic data. JB, JJ, CD, MK, MD-K, CD-K, SM, EC, RN, MB, MS, AV, and DS critically revised the manuscript. All authors have seen and approved the final version of the manuscript being submitted.
