# Supplementary material for: The effect of musicality on language recovery after awake glioma surgery
Source: Front Hum Neurosci. 2023 Jan 10;16:1028897. doi: 10.3389/fnhum.2022.1028897 (PMC9873262; doi:10.3389/fnhum.2022.1028897)
Supplement: Supplementary file 1 [file Data_Sheet_1.docx]

**Appendix A.**

For musicality classification underlying **Table A** was filled in after telephone **questionnaire B**.

This was based on the MEC criteria which defines a musician based on years of musical training and intensity.

**Table A**

Definition of the group labels based on the questionnaire provided.

| Group | I: Non-musicians | II: Amateur musicians (non-playing) | III: Amateur musicians (playing) | IV: Trained musicians (non-playing) | V: Trained musicians (playing) |
| --- | --- | --- | --- | --- | --- |
| Conditions |  |  |  |  |  |
| Playing an instrument?^1^ | *No (0p)* | *Formerly (1p)* | *Yes (2p)* | *Formerly (1p)* | *Yes (2p)* |
| Lessons? | *I do not play an instrument (0p)* | *1-5 years (1p)* | *1-5 years (1p)* | *≥ 6 years (2p)* | *≥ 6 years (2p)* |
| Years of playing? ^2^ | *<1 year (0p)* | *1-10 years (1p)* | *1-10 years (1p)* | *≥ 11 years (2p)* | *≥ 11 years (2p)* |
| Hours of playing a week? | *<0.5 hrs/wk on average (0p)* | *0,5-2 hrs/wk on average (1p)* | *0,5-2 hrs/wk on average (1p)* | *≥ 2,5 hrs/wk on average (2p)* | *≥ 2,5 hrs/wk on average (2p)* |
| Points | *0-1 points* | *1-5 points* | *2-6 points* | *6-7 points* | *7-8 points* |

^1^ The first condition is a prerequisite for group formation.

^2^ For singing, having received lessons or in group formation (band/choir) is a prerequisite. For instrumentalists playing an instrument without lessons is allowed.

**Questionnaire B (Dutch language)**

*Onderstaande vragen gaan over de periode tot aan de operatie (T1)*

1. Zingt u (koor, band, individueel, opleiding)?

a. Ja

b. Nee

c. Voorheen, maar nu niet meer

2. Bespeelt u één of meerdere instrumenten (excl. zang)?

a. Ja

b. Nee

c. Voorheen, maar nu niet meer

Indien tweemaal b. (groep I: non-musicians) hoeft de patiënt de volgende vragen niet meer te beantwoorden.

3. Heeft u les gehad voor de instrumenten die u bespeelt (incl. zang)?

a. Ja

b. Nee, ik ben autodidact

Indien b. hoeft de patiënt vraag 4, 11 en 12 niet meer te beantwoorden.

4. Hoelang heeft u les gehad voor de instrumenten die u bespeelt/bespeelde (incl. zangles)?

a. 1 tot 5 jaar

b. 6 jaar of langer

5. Heeft u in groepsformatie gespeeld/gezongen (band, koor), zo ja hoe lang?

a. Nee

b. 1 tot 5 jaar

c. 6 jaar of langer

6. Hoelang heeft u in totaal uw instrumenten bespeeld (inclusief zang)?

…………………………………………………… jaar

7. Hoeveel tijd heeft u gemiddeld per week besteed aan het spelen van uw instrument (inclusief zang)?

…………………………………………………… uur

8. Welke instrumenten bespeelt/bespeelde u (hoofdinstrument eerst)?

……………………………………………………

9. Op welke leeftijd heeft u de instrumenten leren bespelen (hoofdinstrument eerst)?

……………………………………………………

10. In welke periode van uw leven heeft u instrumenten bespeeld (bijv. tussen 5 en 10 jaar of tussen 25 en 33 jaar) (hoofdinstrument eerst)?

……………………………………………………

11. Hoe frequent heeft u les gehad voor de instrumenten die u bespeelt/bespeelde?

a. 3 of meer keer per week

b. 2 keer per week

c. 1 keer per week

d. 1 keer per twee weken

1. e. 1 keer per drie weken
2. f. 1 keer per maand

12. Heeft/had u groepsles of privéles?

a. Groepsles

b. Privéles

c. Een combinatie

13. Heeft u van bladmuziek leren spelen?

a. Ja

b. Nee

*Onderstaande vragen gaan over de periode ná de operatie (T2/T3)*

14. Wanneer bent u na de operatie weer begonnen met het spelen van uw instrument(en)/zingen?

a. Direct na de operatie

b. Na 4 weken

c. Na 3 maanden

d. Na 6 maanden

e. Na 9 maanden

f. Na 12 maanden

g. Ik heb niet meer gespeeld/gezongen

Indien g. hoeft de patiënt de volgende vraag niet meer te beantwoorden.

15. Hoeveel tijd heeft u in het jaar na de operatie gemiddeld per week besteedt aan het bespelen van uw instrument(en)?

[open question] uur
