# Supplementary material for: The effect of musicality on language recovery after awake glioma surgery
Source: Front Hum Neurosci. 2023 Jan 10;16:1028897. doi: 10.3389/fnhum.2022.1028897 (PMC9873262; doi:10.3389/fnhum.2022.1028897)
Supplement: Supplementary file 2 [file Data_Sheet_2.docx]

**Appendix B. Technical report for volumetric measurements**

**
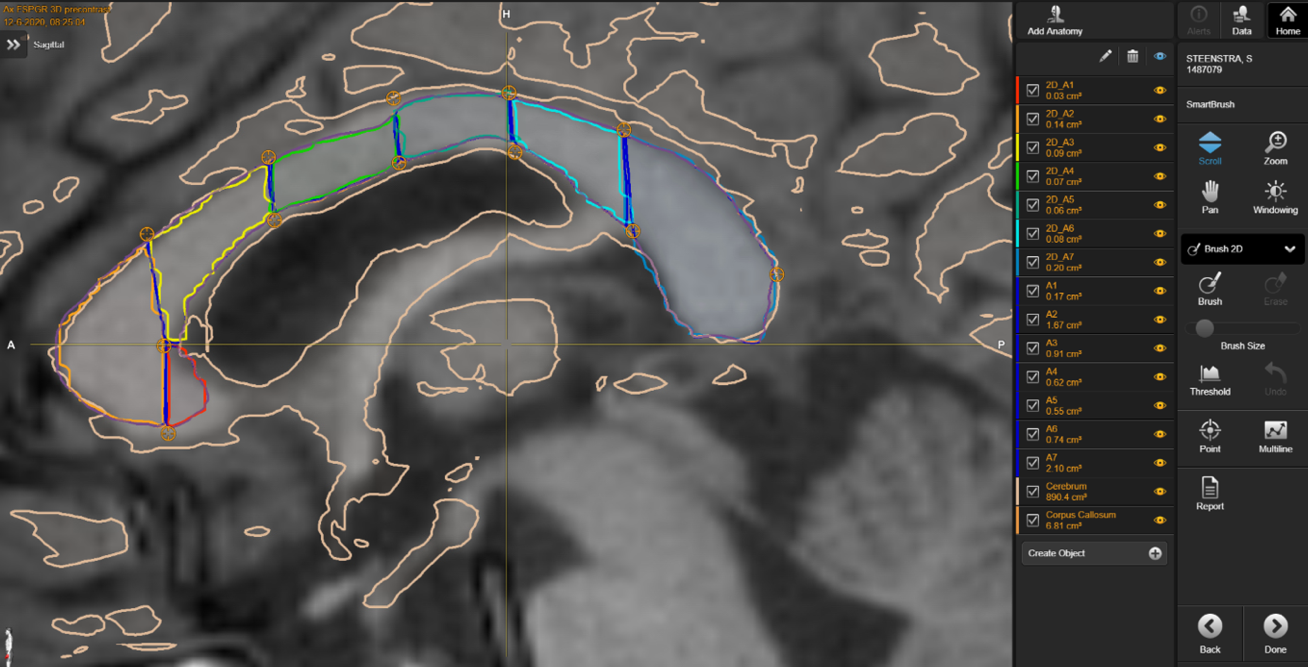
**To measure the size of the corpus callosum we analyzed the most recent structural brain magnetic resonance imaging (MRI: 1.5 or 3.0 Tesla GE Healthcare) before the awake craniotomy, using <1.0mm slide with T1 weighted imaging parameters. Two researchers (P.K. / J.B.), blinded for the outcome on musicality at the time of measurement, first divided the corpus callosum in 7 subregions according to the Witelson classification (see below). Afterwards, volumes (in cubic centimeters/cm^3^) for each subregion were measured with Brainlab’s Synthetic Tissue Model (Brainlab Digital OR, Germany, München). See 1A for the technical report on Brainlab’s Synthetic Tissue Model.

**
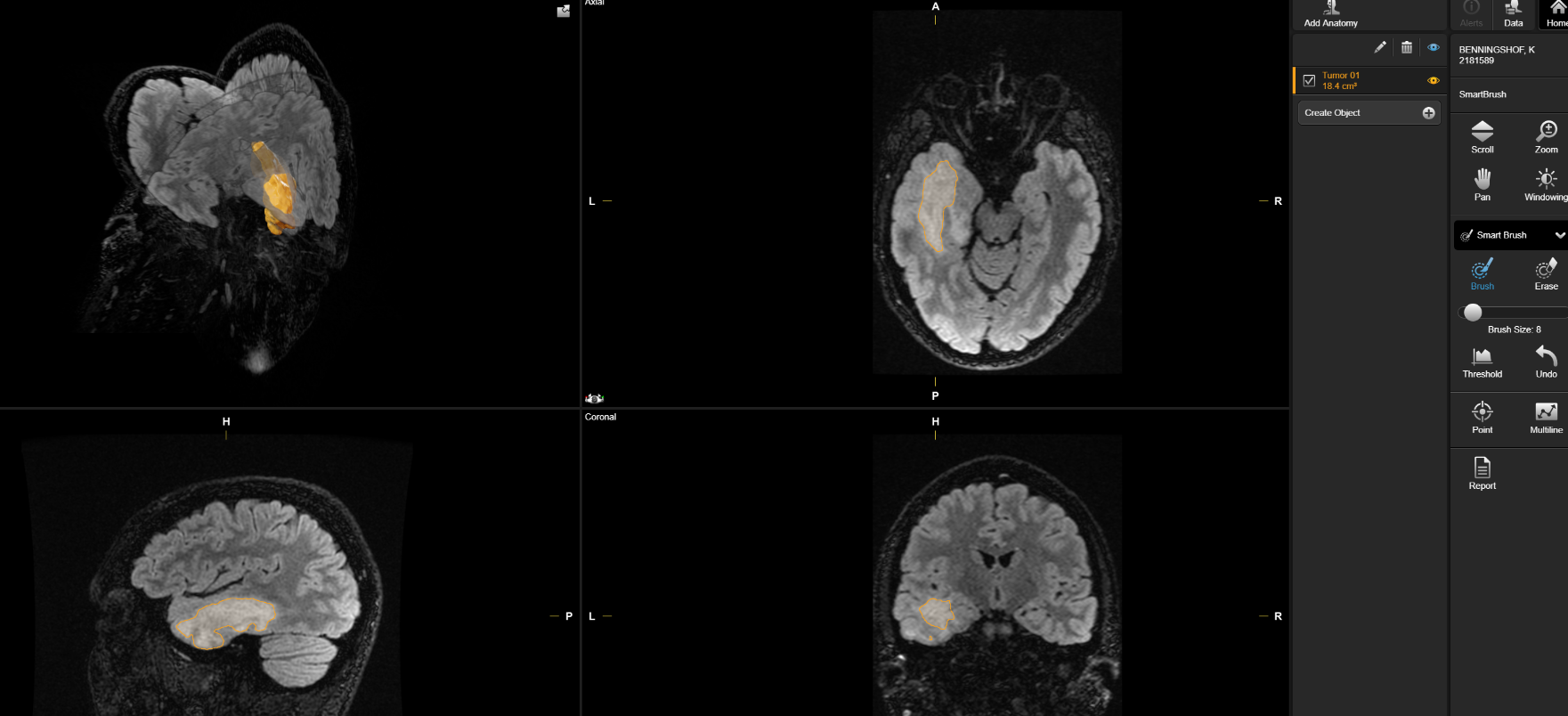
**For the volume lesion analysis, we used the pre-operative coronal, sagittal and transversal T2 weighted FLAIR MRI images and conducted volumetric analysis with Brainlabs’ smart brush (see below for an example).

See 1B for the technical report of Brainlabs’ smartbrush function.

**1B technical report Brainlab’s Synthetic Tissue Model**

**Introduction**

In general, automatic image segmentation applications are able to identify anatomical or pathological structures in medical image datasets. Typically, a fixed atlas is elastically registered to a patient scan and used to label each voxel according to specific anatomies defined in the atlas a priori. Such a fixed atlas, however, cannot fully account for the patients individual anatomy and may also show different image characteristics compared to the patient scan, which limits segmentation accuracy. Therefore, Elements Segmentation introduces a novel approach whereby a Synthetic Tissue Model is employed to simulate the patients anatomy and to generate an individualized, patient-specific atlas, showing same imaging characteristics as the analyzed image set. The Synthetic Tissue Model is a core technology in a variety of Brainlab applications and facilitates reliable segmentation of license-specific structures.

**
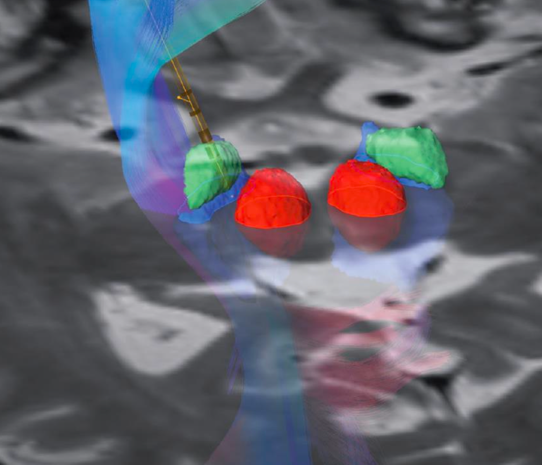
**

**Fig. 1** Representative T2-weighted MRI used for trajectory planning in Deep Brain Stimulation (DBS) with automatic segmentation of red nucleus (red), subthalamic nucleus (green) and substantia nigra (blue). The 3D reconstruction of segmented structures and a model of an implanted lead as well as DTI motor fibers facilitate DBS and support the programming through visualization of the electrode contact position and orientation relative to target structures and other structures in the functional environment^1^.

**Intro**: The Brainlab Synthetic Tissue Model is an universal atlas, which means that it is modality-independent and flexibly adapts to patient anatomy. The model represents a digital twin of patients and serves as the basis for Elements Segmentation – a software module that is part of the Brainlab Elements planning suite. This segmentation application is available with different licenses for segmentation of structures in different anatomical regions (e.g. Cranial, Spine, Basal Ganglia). Depending on the license, the frontend application labeled ‘Anatomical Mapping’ is integrated into specific clinical workflows and used to trigger the background calculation and subsequent visualization and review of automatically segmented objects.


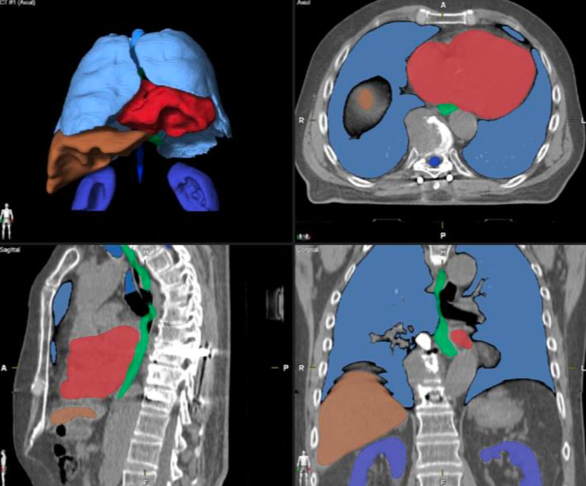
**Fig. 2** Organs-at-risk segmentation in clinical CT data used for planning of radiotherapy (RT) and acquired after surgery. Elements Segmentation software shows the results in ACS views (axial, coronal, sagittal) with an additional 3D model of segmented structures (upper left panel). Expert review demonstrated high clinical acceptance for lungs, kidneys, trachea, heart, liver, spinal cord, and that this segmentation tool provides timesaving, reduced inter- and intra-user variability and therefore higher consistency in RT contouring procedures^2^.

The ‘Anatomical Mapping’ application is intended to support the contouring of organs (at risk) in RT treatments and image-guided or frame-based surgeries (Fig. 1, Fig. 2). Additionally, image recognition by means of the Synthetic Tissue Model supports patient-specific and indication-specific visualizations as well as advanced data post-processing in many Brainlab applications – contributing to the understanding of individual patient anatomy. For instance, tissue recognition is used for more realistic views in ‘Elements Viewer 3D’. An example for post-processing is elastic co-registration of images, which is used for brainshift simulation with ‘Elements Virtual iMRI Cranial’ and patient positioning correction with ‘Elements Curvature Correction Spine’.

**Method**: Each voxel of the Synthetic Tissue Model is labelled with meta information (e.g. tissue class and related elasticity and density) and mapped to the analyzed dataset to segment the patient’s MRI and CT scans. Segmentation results for cranial structures are generally obtained based on MRI. However, some cranial structures are also available for CT. For segmentation of extracranial organs, CT images are required. In order to facilitate the mapping, i.e. registration of the Synthetic Tissue Model to the patient dataset, the model is flexibly adapted in two steps:

• *Anatomy simulation*. After initial body part detection, specific anatomical features of the model are determined to reflect the individual patient anatomy, e.g. detection of space- occupying lesions and estimation of ventricle sizes (cranial), or determination of arm positions and diaphragm location (extracranial).

• *Modality simulation.* The anatomically adapted model is converted into a gray-scale image volume, imitating the characteristics of the analyzed patient scan. Therefore, the modality is first detected and then tissue-class specific gray value simulation is applied, resulting in a patient-specific atlas with e.g. same image contrast as visible in the analyzed CT, T1-, T2-weighted MRI, FLAIR, CISS or SWI dataset.

Due to the flexibility of the Synthetic Tissue Model, multiple CT or MRI scans, acquired with the same frame-of-reference, are exploited simultaneously in terms of image information:

- •  *Cluster registration*. In general, up to six scans acquired in one imaging study can be considered, but need to be acquired in the same body region, with the same frame-of- reference and within a time period of 30 minutes. The scans are iteratively registered to the set of modality-specific atlases (of the Synthetic Tissue Model) while the matching of individual structures or regions is accomplished by individual weighting of each atlas-to-scan registration depending on the corresponding scan properties. In MRI, different scans acquired with different sequence protocols and varying image contrasts are used to facilitate cranial segmentation. For example, T2-weighted MRI showing higher contrast in the basal ganglia region compared to T1- weighted MRI is primarily used to determine the cluster registration for this region. In CT, multiple scans with e.g. different FOVs and reconstructions are used to improve extracranial image segmentation. In order to ensure consistent and accurate segmentation results for the data cluster, motion correction of relevant patient scans is performed in advance.
- •  *Post*-*processing.* After registration of the adapted Synthetic Tissue Model to one or more datasets, methods like shape- preserving smoothing or image intensity-based registration optimization are applied to optimize the segmentation. For image intensity re-registration the patient scan selected by the user in the ‘Anatomical Mapping’ application serves as a basis. Consequently, segmentations using different patient scans within a cluster may yield different segmentation results although based on one unique atlas registration.

**Validation**: The automatic segmentation structures have been evaluated quantitatively and qualitatively, and revalidations are continuously performed for each new version of the algorithm. For the quantitative evaluation, a pool of representative patient data is manually segmented by physicians and compared to the automatic segmentation results. The comparison is based on different metrics quantifying the similarity between automatic segmentation and reference structures (e.g. DICE coefficient and Hausdorff distance). Qualitative evaluation is conducted by visual inspection of representative automatic segmentation results by clinical experts with vast experience in respective medical fields.

**Examples of clinical application**: Previously reported literature show that Elements Segmentation Spine can be used to provide timesaving segmentation of organs-at-risk in spinal metastasis and head-neck cancer treatment planning^2,3^. In addition, automatic segmentation of vertebrae is facilitated by augmented reality applications and intraoperative ultrasound in intradural spinal tumor surgery in order to enhance image interpretation and thereby to support the surgeon during the surgical procedure^4,5^. For DBS, Elements Segmentation Basal Ganglia enables automatic segmentation of the subthalamic nucleus (STN). It was shown that automatic STN segmentation serves as viable starting point to improve and visualize the patient-specific targeting strategy with similar accuracy as provided by non-clinically approved research tools^1,6^. Recently introduced elastic image fusion techniques employ cranial segmentation results to build realistic digital patient models in order to enable robust cranial distortion and spinal curvature correction^7,8^ or to perform brainshift simulation intraopertively^9^. These technologies potentially improve accuracy of planning and navigation of neurosurgical interventions.

**Summary**: Elements Segmentation enables automatic image segmentation by means of a flexible Synthetic Tissue Model, which is individually adapted to the patient’s MR or CT scan. This facilitates the labelling and subsequent outlining of anatomical structures such as organs-at-risk in medical images. The set of available structures is defined by the purchased license, like Elements Segmentation Cranial, Spine or Basal Ganglia, and is growing with successive software releases. The list of currently available structures and supported modalities is given below (available as of February 2020).

**1B. technical report Brainlab’s smartbrush function**

Smartbrush is a semi-automated tool for segmentation, based on a region growing algorithm, a standard technique in medical image processing. Segmentation with the new Smartbrush is started by marking some points inside the desired area. Within an automatically determined ROI around these points, a region-growing algorithm is performed which computes the final 2D-segmentation. This method of segmentation can then either be conducted for each slice or by the 3D-interpolation of the program itself. For the interpolation method the user segments the tumor in one slice, which is ideally as central as possible. After that, segmentation of the desired structure in a plane perpendicular to the segmented slice is performed. The 3D-interpolation automatically detects the three-dimensional ROI and segments the area three-dimensionally with a region-growing algorithm. For final segmentation of the tumor, smoothing is applied to the created object. If necessary, the result of the 3D-interpolation can quickly be adjusted manually. The change to one slide is then interpolated to the complete object to improve the result of the whole segmented structure.

This method can be used not only to segment hard tissue, but also to segment other anatomical regions like the orbit and use this data to plan reconstruction.
Additionally, already segmented structures can be modified using the conventional smart shaper by elastic deformation of the selected object in a selected range.^10, 11^

**References**

1. Reinacher et al. Oper Neurosurg (Hagerstown). 2019 Mar 12

2. Wittenstein et al. Strahlenther Onkol. 2019 Apr 29
3. Daisne and Blumhofer. Radiat Oncol. 2013 Jun 26;8:154
4. Saß et al. World Neurosurg. 2019 Jul 31

5. Carl et al. Acta Neurochir (Wien). 2019 Jul 12
6. Polanski et al. accepted in Stereotact Funct Neurosurg. 2020
7. Gerhardt et al. Clin Neurol Neurosurg. 2019 Jun 10
8. Rashad et al. Int J Med Robot. 2019 Jun;15(3):e1991
9. Riva et al. Oper Neurosurg (Hagerstown). 2019 Jul 25
10. Huber T et al. Clin Neuroradiol. 2017

11. Rana M et al. J Craniomaxillofac Surg. 2015
